# Effects of crowding on the three main proteolytic mechanisms of skeletal muscle in rainbow trout (*Oncorhynchus mykiss*)

**DOI:** 10.1186/s12917-020-02518-w

**Published:** 2020-08-17

**Authors:** Cristián A. Valenzuela, Claudia Ponce, Rodrigo Zuloaga, Pamela González, Ruben Avendaño-Herrera, Juan A. Valdés, Alfredo Molina

**Affiliations:** 1 Laboratorio de Biotecnología Molecular, Facultad de Ciencias de la Vida, Universidad Andrés Bello, 8370146 Santiago, Chile; 2 Laboratorio de Genética e Inmunología Molecular, Instituto de Biología, Pontificia Universidad Católica de Valparaíso (PUCV), Valparaíso, Chile; 3 Interdisciplinary Center for Aquaculture Research (INCAR), 4030000 Concepción, Chile; 4 Laboratorio de Patología de Organismos Acuáticos y Biotecnología Acuícola, Universidad Andrés Bello, 2520000 Viña del Mar, Chile; 5 Centro de Investigación Marina Quintay (CIMARQ), Universidad Andrés Bello, 2340000 Quintay, Chile

**Keywords:** Fish, Stress response, Autophagy, Ubiquitin-proteasome, Calpain/calpastatin

## Abstract

**Background:**

Skeletal muscle is one of the tissues most affected by stress conditions. The protein degradation in this tissue is vital for the supply of energy mediated by different proteolytic pathways such as the ubiquitin-proteasome (UPS), autophagy-lysosome (ALS) and the calpain/calpastatin system (CCS). Nevertheless, the regulation of this proteolytic axis under stress conditions is not yet completely clear. Chile is the main producer of rainbow trout (*Oncorhynchus mykiss*) in the world. This intensive fish farming has resulted in growing problems as crowding and stress are one of the major problems in the freshwater stage. In this context, we evaluated the crowding effect in juvenile rainbow trout kept in high stocking density (30 kg/m^3^) for 15, 45 and 60 days, using a control group of fish (10 kg/m^3^).

**Results:**

Plasmatic cortisol and glucose were evaluated by enzyme immunoassay. The mRNA levels of stress-related genes (*gr1, gr2, mr, hsp70, klf15* and *redd1*), markers of the UPS (*atrogin1* and *murf1*) and CCS (*capn1, capn1, cast-l and cast-s*) were evaluated using qPCR. ALS (LC3-I/II and P62/SQSTM1) and growth markers (4E-BP1 and ERK) were measured by Western blot analysis. The cortisol levels increased concomitantly with weight loss at 45 days of crowding. The UPS alone was upregulated at 15 days of high stocking density, while ALS activation was observed at 60 days. However, the CCS was inactivated during the entire trial.

**Conclusion:**

All these data suggest that stress conditions, such as crowding, promote muscle degradation in a time-dependent manner through the upregulation of the UPS at early stages of chronic stress and activation of the ALS in long-term stress, while the CCS is strongly inhibited by stress conditions in the rainbow trout muscle farmed during freshwater stage. Our descriptive study will allow perform functional analysis to determine, in a more detailed way, the effect of stress on skeletal muscle physiology as well as in the animal welfare in rainbow trout. Moreover, it is the first step to elucidate the optimal crop density in the freshwater stage and improve the standards of Chilean aquaculture.

## Background

Chile is the main producer of rainbow trout (*Oncorhynchus mykiss*) in the world. This farming activities typically involve three distinct stages, namely, fresh water, smoltification (commonly in brackish waters), and life in the sea; and life in the sea, whose main objective to produce intensively with a lower economic cost. Due to the increase in demand for protein sources for human consumption, the salmon farming industry has had to adopt inappropriate practices to supply this demand, such as the use of high densities (< 30 kg/m^3^) of culture during the freshwater stage. In fact, the freshwater phase is unregulated on aspects such as fish density against the primary pathogens of this stage, such as *Flavobacterium psychrophilum*. This lack of regulation as compared to the seawater stages (10 to 15 kg/m^3^ depending on the farmed species, i.e. trout or salmon) of the salmon lifecycle is surprising, increasing the vulnerability of the rainbow trout, especially since Chilean regulations on seawater rearing can often be stricter than other salmonid-producing countries, including Norway, Scotland, Ireland, and Canada [1]. Moreover, fish movements are an integral part of the Chilean salmon farming production cycle and has been recognized as a major risk for the introduction and spread of highly infectious diseases in fish. These practices cause a perturb in the animal welfare due to increase the stress, negatively impacting several different biological processes such as growth muscle as well as raising susceptibility of rainbow trout to bacterial and/or viral infection.

Skeletal muscle is the biggest tissue in fish and the most important protein source for human consumption; therefore, it is the most significant product for the fish industry [2]. The growth of muscle depends on the balance between anabolic and catabolic processes, and when the rate of degradation is greater than synthesis, the phenomenon of muscle atrophy is triggered [3]. Muscle atrophy derived from excessive proteolysis (catabolism) can lead to an inhibition of muscular growth [4]. In particular, three main mechanisms involved in the degradation of muscular protein are the ubiquitin-proteasome system (UPS), the autophagy-lysosome system (ALS) and the calpain/calpastatin system (CCS) [5]. In fish, these processes have a key role in protein turnover and energy production [6].

Protein degradation by the UPS consists of the polyubiquitination of target proteins as a substrate for degradation; enzymes such as E1, E2 and E3 participate in this process and are key components in this mechanism [7]. In this system, the proteins are labeled for ubiquitination, then recognized by the 26S proteasome complex and are degraded into oligopeptides [8]. However, in fish such as rainbow trout this system is responsible for only 17% of the protein degradation of myotubes [9]. The two specific markers for UPS in fish muscle are known as MurRF1 (Muscle RING-finger protein 1) and Atrogin1 [10, 11]. On the other hand, the ALS is responsible for more than 30% of total protein degradation of the skeletal muscle cells in fish [9]. In this process, portions of cytoplasm and cell organelles are sequestered into vesicles called autophagosomes, which then fuse with lysosomes or vacuoles for enzymatic breakdown [12]. At the molecular level, this mechanism is mediated by the conversion of LC3-I (Microtubule-associated protein 1A/1B-light chain 3) to the active form LC3-II and the degradation of P62/SQSTM1 protein content [13]. Finally, the CCS is involved in protein degradation during pre- and post- mortem events. This system has two main components: calpain and calpastatin, where calpains (μ- and m-calpain) are Ca^+ 2^-dependent cysteine proteases, while calpastatin is the specific inhibitor of the calpain protease activity [14]. The function of this mechanism is mainly the degradation of muscle Z-discs [15].

In general terms, these three mechanisms can be modulated by different conditions and factors, stress being the most common and important for the fish aquaculture industry. Precisely, several routine conditions, such as confinement or crowding, handling, and vaccinations, can trigger the stress response, negatively impacting the productivity of this economic sector. For example, it is well documented that stress can inhibit the somatic growth of muscle in fish by downregulating the expression of growth-related genes in salmonids [16].

The stress response begins with activation of the hypothalamus-pituitary-interrenal (HPI) axis and the release of cortisol, the principal corticosteroid (CS) in fish [17]. After this primary stress response, cortisol can interact with the glucocorticoid receptors (GRs) and the mineralocorticoid receptor (MR) to promote the modulation of expression of different target genes of the GR/MR [18]. Finally, biological processes, such as swimming ability, pathogen resistance, and growth rate, can be affected by stress in a response known as the tertiary stress response [19].

Several external factors can trigger a stress condition, such as high stocking density and food deprivation, and can directly and indirectly affect muscle growth, consequently promoting muscular atrophy in fish [20, 21]**.** For example, the stress triggered by high stocking densities can activate different mechanisms involved in muscular atrophy such as the UPS in grass carp (*Ctenopharyngodon idellus*) [22]. In addition, in juvenile fine flounder (*Paralichthys adspersus*) subjected to 4 and 7 weeks of chronic stress, both the UPS and ALS were upregulated in a nonoverlapping way [4].

Despite these antecedents, little is known about the modulation of the UPS, ALS and CCS after long-term crowding stress in rainbow trout. In this context, the aim of this study was to analyze in detail these three processes at the molecular level in an integrative and descriptive manner using rainbow trout as a model system. For this, juvenile rainbow trout (~ 8 g) were subjected to 15, 45 and 60 days at two different densities: low density (LD) was set at 10 kg/m^3^ and high density (HD) was set at 30 kg/m^3^. Several UPS-related genes, ALS markers and CCS transcripts were assessed. Considering that Chile is the main producer of rainbow trout worldwide, studies like this are essential to have an approximation on the adequate culture density of salmonids farmed during the freshwater stage.

## Results

### Morphometrical and stress parameters

**Fig. 1:**
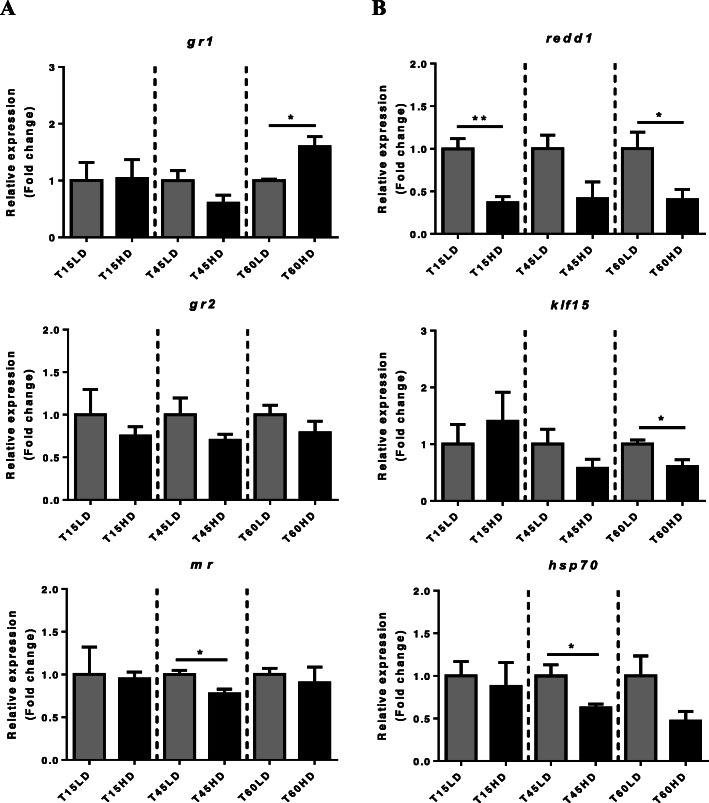
High density-induced stress on the cortisol signaling pathway in skeletal muscle. Relative expression (fold change) of (**a**) corticosteroid receptors *gr1, gr2* and *mr* (**b**) GR target genes: *redd1*, *klf15*, and *hsp70* normalized with *fau* and *β-actin*. Results are shown as the means ± SEM (*n* = 5 per group). LD, low stocking density; HD, high stocking density. Significant differences are indicated with a line (* = *P* < 0.05; ** = *P* < 0.01)

To evaluate the effect of crowding stress on morphological and molecular mechanisms of fish growth, juvenile trout were subjected to 15, 45 and 60 days of treatment at two different densities (high [HD] and low [LD]). Body weight, total length and condition factor (K) in each experimental group are shown in Table 1. Significant differences where observed only in body weight at 45HD (*P* < 0.05). In the HD groups, only at 45 days had plasma cortisol levels increased significantly compared with the LD group at the same time; however, a tendency can be observed at the other experimental times. There were no significant differences in plasmatic glucose (GLU) and total protein (TP) between LD and HD in the entire trial (Table 2). The corticosteroid receptors (CRs) *gr1*, *gr2* and *mr* were assessed, where only *gr1* was upregulated at 60HD, while *gr2* did not change and *mr* was downregulated at 45HD (*P* < 0.05) (Fig. 1a). The CR target genes *redd1* and *klf15* (*P* < 0.01, *P* < 0.05) and the stress marker *hsp70* were downregulated under HD conditions (*P* < 0.05) (Fig. 1b).

**Table 1:** Values (± standard deviation) of the morphological biometric parameters of rainbow trout (*O. mykiss*) at 15, 45 and 60 days of treatment. The results are shown as the means ± SEM (*n* = 5 per group) Significant differences shown with a line (* = *P* < 0.05)

	15 Days		45 Days		60 Days	
	LD	HD	LD	HD	LD	HD
Body length (cm)	9 ± 0.46	9.6 ± 0.24	10.2 ± 0.43	9.5 ± 0.35	11.79 ± 1.8	11.62 ± 1.17
Body weight (g)	8.74 ± 1.34	9.92 ± 0.45	12. 89 ± 1.42	9.81 ± 0.379 *	10.1 ± 0.51	10.5 ± 0.4
Condition factor (K)	1.18 ± 0.07	1.12 ± 0.05	1.21 ± 0.05	1.15 ± 0.10	1.19 ± 0.07	1.16 ± 0.12

**Table 2:** Plasma levels of cortisol, glucose and total proteins of rainbow trout (*O. mykiss*) at 15, 45 and 60 days of treatment. The results are shown as the means ± SEM (*n* = 5 per group). Significant differences are shown with a line (* = *P* < 0.05)

	15 Days		45 Days		60 Days	
	LD	HD	LD	HD	LD	HD
Glucose (GLU) (UL¯^1^)	173.68 ± 9.93	189.59 ± 7.43	193.85 ± 3.79	191.16 ± 4.86	187.82 ± 3.73	192.6 ± 12.47
Cortisol (mmol L¯^1^)	172.79 ± 35.96	346.79 ± 69.52	692.32 ± 182.07	1314.85 ± 99.38*	482.13 ± 108.07	1045.73 ± 474.71
Total protein (TP) (ng μL¯^1^)	4.94 ± 0.18	4.68 ± 0.16	3.85 ± 0.01	3.46 ± 0.24	4.85 ± 0.07	5.1 ± 0.12

### Muscle growth signaling pathways

**Fig. 2:**
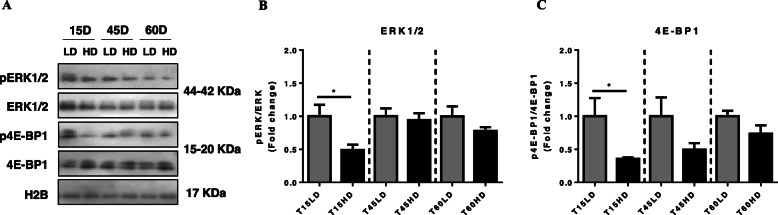
Effect of high density-induced stress on the IGF-1 system. **a** Representative Western blots of key genes of IGF-1 system, ERK and p4E-BP1, in phosphorylated and total forms, with H2B as the loading control. **b** Fold change of pERK/ERK ratio (**c**) Fold change of p4E-BP1/4E-BP1 ratio. Results are shown as the means ± SEM (*n* = 5 per group). LD, low stocking density; HD, high stocking density. Significant differences are indicated with a line (* = *P* < 0.05)

Activation of the IGF-1/TOR and IGF-1/ERK signaling pathways was evaluated. The ratios of both p4E-BP1/4E-BP1 and pERK/ERK were significantly downregulated at 15HD (*P* < 0.05) (Fig. 2a, b and Supplementary Figure S2).

### Ubiquitin-proteasome system gene expression

**Fig. 3:**
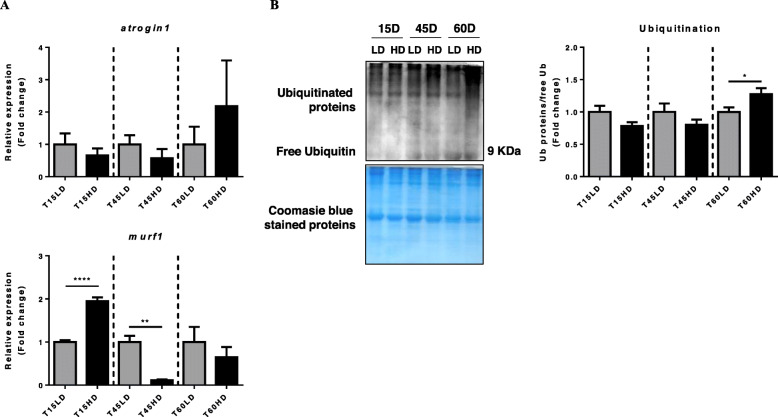
Effect of high density-induced stress on the ubiquitin-proteasome system. **a** Relative expression (fold change) of key genes of the ubiquitin proteasome system, *atrogin1* and *murf1*, normalized with *fau and β-actin*. **b** Representative Western Blot of Ubiquitinated proteins. Coomassie blue-stained loading control proteins. Fold change of ubiquitinated proteins/free ubiquitin ratio. Results are shown as the means ± SEM (n = 5 per group). LD, low stocking density; HD, high stocking density. Significant differences are indicated with a line and an asterisk (* = *P* < 0.05; ** = *P* < 0.01; **** = *P* < 0.0001)

Then, the gene expression of the “atrogenes” *atrogin1* and *murf1* was assessed. The mRNA level of *murf1* was increased at 15HD (*P* < 0.0001) and then decreased at 45HD (*P* < 0.01), while *atrogin1* mRNA levels did not change (Fig. 3a). Additionally, protein ubiquitination was observed and showed a significant increase at 60HD (*P* < 0.05) (Fig. 3b and Supplementary Figure S3).

### Autophagy-lysosome system molecular markers

**Fig. 4:**
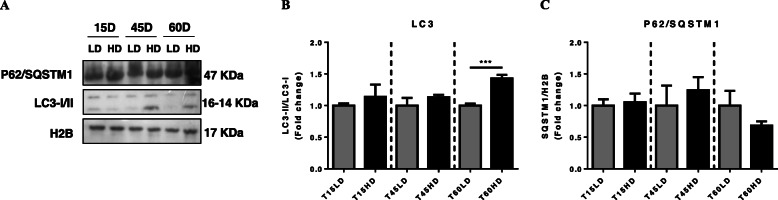
Effect of high density-induced stress on the autophagy-lysosome system. Representative blots of the content of key proteins of autophagy system (**a**) LC3 II/I and (**b**) P62/SQSTM1 normalized with H2B. The results are shown as the means ± SEM (*n* = 5 per group). LD, low stocking density; HD, high stocking density. Significant differences are indicated with an asterisk (*** = *P* < 0.001)

The autophagy proteins LC3 and P62/SQSTM1 were analyzed. The LC3-II/LC3-I protein content ratio was significantly upregulated only at 60HD (*P* < 0.001), whereas P62/SQSTM1 protein content did not change during the entire trial (Fig. 4a, b and Supplementary Figure S4).

### Calpain/calpastatin system expression and activity

**Fig. 5:**
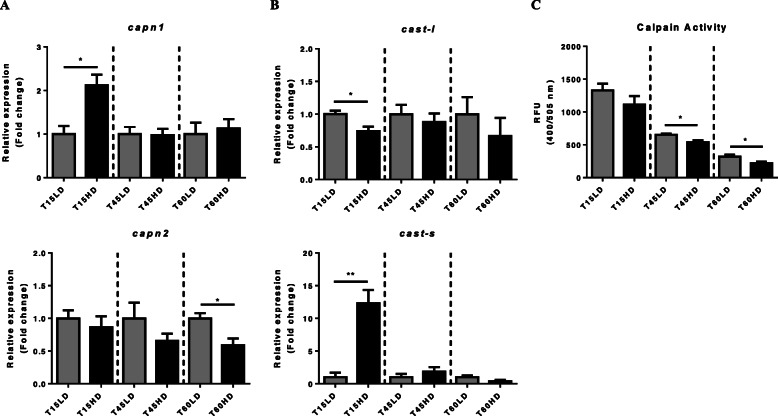
Effect of high density-induced stress on the Calpain/calpastatin system. Relative expression (fold change) of (**a**) *calpain 1–2* and (**b**) the specific inhibitor *calpastatin* (short and long isoforms). Each gene was normalized using *fau* and *β-actin*. **c** Calpain enzymatic activity in relative fluorometric units (RFU). The results are shown as the means ± SEM (*n* = 5 per group). LD, low stocking density; HD, high stocking density. Significant differences are indicated with a line and an asterisk (* = *P* < 0.05; ** = *P* < 0.01)

Finally, the calpain/calpastatin system was evaluated. At the transcriptional level, *capn1* was significantly upregulated at 15HD (*P* < 0.05) while *capn2* mRNA levels decreased significantly at 60HD (*P* < 0.05) (Fig. 5a). On the other hand, *cast-l* was downregulated at 15HD (*P* < 0.05) and *cast-s* transcript levels increased (*P* < 0.01) in the same experimental time frame (Fig. 5b). However, calpain activity was progressively downregulated during the entire trial (*P* < 0.05), reaching its lowest level at 60HD (Fig. 5c).

## Discussion

Crowding stress can lead to an inhibition of muscle growth in fish, through muscular atrophy modulation [4, 21]. Despite this, little is known about the molecular mechanisms involved in the promotion of muscle atrophy under stress conditions and the dynamic over time of these processes in rainbow trout, an aquatic species of great commercial interest worldwide. In this work, we showed the impact of crowding conditions on rainbow trout skeletal muscle through the molecular profile of the three main proteolytic mechanisms involved in muscular degradation. Classically, a complete stress response is represented by an increased level of cortisol and glucose in plasma, followed by the upregulation of the mRNA levels of several GR target genes such as *klf15* and *redd1* [23]. This modulation of gene expression can finally lead to an inhibition of muscle growth and other physiologic processes such as swim performance, reproduction and immune system function [19].

As a primary stress response, cortisol plasma levels increased significantly only at 45HD. However, over the entire trial, a tendency of cortisol levels to increase in both LD and HD groups was observed. Although it is well known that cortisol levels usually increase hours after exposure to a stressful condition in rainbow trout, zebra fish (*Danio rerio*) and gilthead seabream (*Sparus aurata*) [24], other studies have shown that cortisol levels increase significantly after 30 days of crowding-stress in rainbow trout [21]. Consequently, cortisol release can be associated with upregulation of the mRNA levels of the corticosteroid receptors *gr1, gr2*, and *mr* [25] and the target genes of these receptors *klf15* and *redd1* [26]. Here, no concomitant increases in cortisol and CR transcript levels were observed, where *gr1* was upregulated only at 60HD, *mr* was downregulated at 45HD, and *gr2* mRNA levels did not change. These results differ from those reported in other studies, where an increase in cortisol levels and CR mRNA levels have been observed within the same experimental time frame, suggesting an upregulation in the transcription of GR genes by cortisol [27]. Despite the upregulation of *gr1*, the mRNA levels of the GR target genes *klf15* and *redd1* were downregulated, suggesting a negative regulation of the pathway associated with KLF15/REDD1 in trout under crowding stress. It is well known that these two molecules are involved in the promotion of atrophy and growth inhibition in fish muscle [4]. Particularly, REDD1 is involved in the inhibition of mTOR, a key molecule in the growth pathway IGF-1/Akt/mTOR [28]. In this study, growth pathways were downregulated at 15HD, where pERK/ERK and p4E-BP1/4E-BP1 ratios were reduced, suggesting a reduction in protein synthesis and, consequently, an inhibition of growth. Similar effects were observed in teleost fish subjected to hypoxia, where it was determined that a decrease in protein synthesis during hypoxia is likely controlled by signaling molecules such as 4E-BP1 [29]. In the case of ERK, under nutritional stress, the phosphorylation of this molecule was downregulated in fine flounder [30]. A similar response was observed in the common carp (*Cyprinus carpio*), where under chronic exposure to fluoride, the ERK pathway was inactivated [31]. These antecedents, together with the results obtained in this work, suggest that the Akt/TOR pathway can be very sensitive to chronic stress conditions in fish at early stages (15 days of treatment).

One of the consequences of muscle growth inhibition can be the activation or promotion of muscle atrophy, which is carried out by different molecular processes such as UPS, ALS and CCS. In the present work, these three mechanisms showed different transcriptional behaviors, where at early time (15 days of crowding), the UPS was upregulated through an increase in *murf1* gene expression. As a primary marker of protein degradation, MuRF1 is a key E3 ligase involved in the control of the half-life of important muscle structural proteins, including troponin I, myosin heavy chains, myosin binding protein C and myosin light chains [32]. In contrast, with our results, low stocking densities promote UPS activity in grass carp (*Ctenopharyngodon idella*), suggesting that fish with better growth performance exhibited higher UPS activity in parallel with the activation of protein synthesis [22]. Despite these antecedents, in the present work we observed a concomitant upregulation of *murf1* and activation of 4E-BP1, suggesting that high stocking density exerted a potent effect on protein degradation and protein synthesis in the early stages of chronic stress (15 days of crowding).

Long term (60 days of high stocking density), only the ALS was upregulated in rainbow trout under crowding conditions. Autophagy constitutes the main mechanism for the degradation of unneeded components such as damaged/nonfunctional organelles and protein aggregates, to maintain the homeostasis of the cell [33]. This cellular mechanism is a dynamic process in which a double-membraned cytoplasmic vesicle, the autophagosome, selectively engulfs damaged proteins, organelles and fractions of cytoplasm and fuses with the lysosome (forming the autolysosome), degrading the sequestered components via lysosomal hydrolases [34]. One of the key molecules involved in the double-membrane vesicle formation is LC3 (Microtubule-associated protein 1A/1B-light chain 3), which is used as a specific marker of autophagy activation [35]. This molecule, in its inactive form LC3-I, is conjugated to phosphatidylethanolamine to form LC3-phosphatidylethanolamine conjugate (LC3-II), which is recruited to autophagosome [36]. Although there are few studies that describe the effect of stress on autophagy in fish, in the last years this process has gained more attention due to its importance and implication in the regulation of cellular homeostasis. For example, Lu et al. (2019) [37] demonstrated that autophagy is involved in the protection of zebrafish under cold temperatures, preventing cell damage. However, in higher vertebrates the effect of stress over the autophagy is well characterized, where dexamethasone, a synthetic glucocorticoid, can upregulate autophagy, thereby promoting mitochondrial clearance [38]. Moreover, the importance of autophagy in skeletal muscle was reviewed by Neel et al. (2013) [39], who described that this process as a metabolic regulator that can be involved in several muscular diseases if it is dysregulated. In a previous work, we suggested that under chronic stress conditions, autophagy can play a cytoprotective role in fish; however, functional assays are required to corroborate this hypothesis.

Finally, we evaluated the expression profiles and activity levels of CCS, one of the most important groups of intracellular proteases in the muscle [40]. These molecules have been characterized in fish and used as markers of flesh quality and texture [14]. Despite this information, only a few reports have shown the transcriptional profile of these molecules under stress conditions, such as starving, where the mRNA levels of the calpains were upregulated [14, 15]. In the present work, we observed a concomitant upregulation of *capn1* and *cast-s*, while *capn2* and *cast-l* were downregulated at 15HD and 60HD, respectively. While an increase in the expression of calpain 1 was observed, the enzymatic activity of these molecules gradually decreased during the experiment. This phenomenon was reviewed in mammals, where increased expression of calpain per se is unlikely to make a significant difference in activity [41]. Even more, less than 10% of total calpain is normally activated in mammalian skeletal muscle [42]. In this sense, chronic stress could negatively modulate activity of the calpains, which, as described in mammals, could be directly associated with the levels of calcium present in the muscle cells. To corroborate this, it is necessary to perform functional assays, which would allow us to quantitatively determine the implication of cytoplasmic calcium in fish stress models.

Scientific support on establishing the most appropriate densities in the farming of rainbow trout during the freshwater stage is essential. Not only for the rapid growth of fish, but also for animal welfare and less susceptibility to bacterial diseases that affect muscle such as *F. psychrophilum* [1]. Precisely, in Chile, there are no regulations associated with farming densities in this stage. In this context, the use of molecular parameters like genes used in the present work could allow define new strategies to improve the freshwater stage production of fish.

## Conclusion

In summary, we descriptively showed the effect of chronic stress due to high stocking densities on the modulation, at the molecular level, of the three main proteolytic mechanisms in the rainbow trout skeletal muscle during freshwater phase. Here, we observed a time-dependent regulation of these mechanisms, where in the early stages of stress, the UPS was upregulated, while ALS was activated during long-term stress. Finally, the CCS was negatively regulated throughout the trial, progressively decreasing in activity until reaching its lowest level at the end of the experiment. The data obtained here could generate new government regulations for the farming of rainbow trout in Chile during freshwater stage and worldwide. In addition, this descriptive study will allow perform functional analysis to determine, in a more detailed way, the effect of stress on skeletal muscle physiology as well as in the animal welfare.

## Methods

### Fish husbandry, experimental design and sampling

Juvenile and sexually immature rainbow trout (7.24 ± 1.35 g body weight [mean ± standard deviation]) from the same farming batch were randomly allocated among six different 8 L plastic tanks with aerated dechlorinated water at 15 ± 1 °C and acclimatized for 1 week before the trial. The fish were fed with commercial pellets (Skretting, Chile) at 1.5% body weight daily and maintained with 12:12 h light:dark of photoperiod. Three tanks contained 10 individuals, corresponding to “low stocking density” (LD) rearing conditions (10 kg/m3), and three tanks contained 30 individuals with “high stocking density” (HD) (30 kg/m3). Rainbow trout from LD and HD tanks were sampled at three experimental points: 15, 45 and 60 days (hereafter mentioned as 15LD, 15HD, 45LD, 45HD, 60LD and 60HD). As the sampling requires removing the fish from each tank for euthanized, which affecting the density (i.e. decreases overcrowding), and it is not convenient to replace with other specimen kept in another pond and conditions. With the aim not to affect the results about the response of rainbow trout to density studied, we consider each density-by-tank combination was only sampled once. Five individual samples per tank (*n* = 5 per treatment) were randomly euthanized using an anesthetic overdose (300 mg/L, BZ-20®, Veterquímica, Chile) at the same local time (1200) (experimental design in Supplementary information Figure S1). Blood from caudal vessel was collected using 1 mL heparinized (10 mg/mL) syringe, then centrifuged at 2000 x g for 5 min at 4 °C to collect plasma, immediately frozen in liquid nitrogen and stored at − 80 °C until they were analyzed. After blood was collected, white myotome skeletal muscle was sampled from all fish, specifically a cross section from the epaxial area. Finally, samples were frozen by liquid nitrogen and stored at − 80 °C until processed for RNA and protein extraction. The conduct of the experiment and outcome assessment were made in the laboratory of Dr. Ruben Avendaño-Herrera (Viña del Mar, Chile) and the data analysis were performed in the laboratory of Dr. Alfredo Molina (Santiago, Chile).

### Growth performance

At the beginning and end of the trial, body weight (g) and total length (cm) of each sampled fish were measured. Also, the condition factor (K) was calculated according previously described formula [30].

### Cortisol and glucose plasma levels

Cortisol and Glucose plasma levels were analyzed by the Enzyme Immunoassay Kit (Cayman, USA) and glucose kit (Valtek Diagnostics, Chile), respectively, following the manufacturer’s recommendations. Both commercial kits were previously validated in teleost fish [43, 44].

### Quantitative real time PCR (qPCR)

Total RNA was isolated from 0.1 g of skeletal muscle following the manufacturer’s recommendations of RNeasy® Mini Kit (Qiagen, USA). RNA was quantified by NanoDrop technology with Epoch Multi-Volume Spectrophotometer System (BioTek, USA). RNA quality was assessed by 1.2% formaldehyde agarose gel electrophoresis containing SafeView™ Classic nucleic acid stain (Abm, Canada). Residual genomic DNA was removed, and 1 μg was used for reverse transcription by Quantitect® reverse transcription kit (Qiagen, USA), according manufacturer’s conditions. qPCR reactions were performed in a MX3000P thermocycler (Stratagene, USA). Each reaction mixture had 6 μl of cDNA (a 40-fold dilution), 0.75 ul of each primer (250 nM) and 7.5 μl of 2× Brilliant® II SYBR® master mix (Stratagene, USA) in a 15 μl final volume. Primers were design using a similar approach that was previously described [4]. The primer sequences and their efficiency used in this study are listed in Table 3. Amplifications were made in triplicate using the following thermal cycling conditions: initial activation 95 °C for 10 min, 40 cycles of 30 s denaturation at 95 °C, 30 s annealing at 56–63 °C, and 30 s elongation at 72 °C. Relative expression analysis was conducted using geNorm software (https://genorm.cmgg.be/) and the results were expressed as fold induction using *β-actin* and *fau* as stable reference genes.

**Table 3:** Primer sequences for qPCR assay, amplicon size, PCR efficiencies, and GenBank accession numbers for the genes used in this study

Gene	Sequence 5′ - 3’		TM (°C)	% E	GenBank code
*40S ribosomal protein S30 (fau)*	Forward	CATTTAGGAGTTGGCGTTGG	60	100%	SRX612429
	Reverse	CCAAGGTTGAAAAGCAGGAG			
*β-actin*	Forward	GCCGGCCGCGACCTCACAGACTAC	65	103.6%	KC888023.1
	Reverse	GCCGGCCGCGACCTCACAGACTAC			
*glucocorticoid receptor 1 (gr1)*	Forward	Teles et al. 2013 [45]	58	101.2%	Z54210.1
	Reverse				
*glucocorticoid receptor 2 (gr2)*	Forward	Teles et al. 2013 [45]	63	99.7%	AY495372.1
	Reverse				
*mineralocorticoid receptor (mr)*	Forward	Teles et al. 2013 [45]	63	102.6%	AF209873.1
	Reverse				
*heat-shock protein 70 (hsp70)*	Forward	CATCGGTGAGTTCAAGCGTA	60	98.30%	PRJNA518130
	Reverse	GCCCTGGTAATGGAGGTGTA			
*regulated in development and DNA damage response 1 (redd1)*	Forward	GGGGGAGGTGTGTCAGAGTA	61	103.5%	XM_021615231
	Reverse	TTACGGGACTGGATGGAGAC			
*krüppel-like factor 15 (klf15)*	Forward	GGAGAGGAGGAAGAGGAGG	60	101.1%	XM_021609837.1
	Reverse	TGTTCAATGGAGCTGGAAGG			
*F-box protein 32 (atrogin1)*	Forward	GAATCTGCGGCTGTCTGTT	61	99.8%	NM_001195177.1
	Reverse	CCTCCTGTTGTCCTTGATGG			
*muscle RING finger 1 (murf1)*	Forward	CAAGAGCATCGAGGAGAACAG	61	97.40%	NC_035094.1
	Reverse	TCCTCTGTCACCACATCATCA			
*calpain 1 (capn1)*	Forward	TCCTTTTGGAAGCCATTTT	56	104.6%	AY573919.1
	Reverse	GGATATTGTGGGGGTTTT			
*calpain 2 (capn2)*	Forward	GAAGGACAAGGATTTGGACG	60	101.8%	AY573920.1
	Reverse	CCTGACAGAGCCTCATAGC			
*calpastatin L (cast-l)*	Forward	CTCAGTAGCCGTGACAA	56	102.2%	AY937407.1
	Reverse	GCTCTTGCCATCCTTATT			
*calpastatin S (cast-s)*	Forward	GATGGGGGAGAGAGATGTCA	62	100%	AY937408.1
	Reverse	ACTGGGCTGTGTCTGCTT			

### Western blotting

The entire protocol in detail for western blotting was previously reported [4], with minor modifications. Briefly, after protein extraction from 0.1 g muscle samples, total protein contents were quantified using Pierce BCA Protein Assay Kit (Thermo Scientific, USA). Twenty-five μg of proteins were resolved in a 7–15% SDS-PAGE and electro-transferred to PVDF membranes with Trans-blot Turbo Transfer System (Bio-Rad, USA). The membranes were blocked using 2% ECL Prime blocking agent (GE Healthcare, UK) for 1 h at room temperature and then incubated with antibodies (phosphorylated, total and secondary antibodies) overnight at 4 °C in shaking. The commercial antibodies and dilutions used in this study are listed in Table 4. After washes, membranes were visualized by ECL Prime Western Blotting Detection Kit (GE Healthcare, UK). Also, membranes were immunoblotted against anti-H2B for loading control (Abcam, UK). Finally, films were scanned for densitometric analysis of protein target bands and showing a representative blot film.

**Table 4:** Types, dilutions, and other features of antibodies used to detect different proteins. The letter in the primary antibody dilution indicates the secondary dilution used

Antibody	Dilution	Brand	Catalog N°
Sequestosome 1 (P62/SQSTM1)	1:1000a	Abcam	91,526
Microtubule-associated protein 1A/1B-light chain 3 (LC3A/B)	1:1000a	Cell Signaling	12,741
Phospho-Extracellular signal-regulated kinases 1/2 (pERK1/2)	1:2000c	Cell Signaling	9106
Extracellular signal-regulated kinases 1/2 (ERK1/2)	1:2000c	Cell Signaling	9107
Phospho-Eukaryotic translation initiation factor 4E-binding protein 1 (p4E-BP1)	1:1000a	Cell Signaling	9459
Eukaryotic translation initiation factor 4E-binding protein 1 (4E-BP1)	1:1000a	Cell Signaling	9452
Ubiquitin	1:2000b	Cell Signaling	3933
Histone 2B (H2B)	1:2000b	Abcam	17,901
Anti-rabbit IgG	1:2000a	Cell Signaling	7074
Anti-rabbit IgG	1:4000b	Cell Signaling	7074
Anti-mouse IgG	1:4000c	Cell Signaling	7076

### Statistical analysis

All data generated were evaluated by two-way ANOVA followed a Tukey’s honestly significant difference (HSD) a-posteriori to determine specific differences between factors (density and time). Data are expressed as mean (*n* = 5 per treatment) ± standard error of mean (SEM), and *p* < 0.05 was considered statistically significant. All analyses were performed using general linear models using Graph Prism 7.0 software (GraphPad Software, Inc., USA).

## Supplementary information

**Additional file 1:** **Figure S1.** Graphical abstract of the experimental design. **Figure S2.** Original western blot images used for growth signaling densitometric analysis. **Figure S3.** Original western blot and gel images used for ubiquitination densitometric analysis. **Figure S4.** Original western blot films scanned for autophagy densitometric analysis.

## Data Availability

The nucleotide sequences used in this study were collected from the National Center for Biotechnology Information (NCBI) GenBank repository. The GenBank accession numbers of all sequences were listed in the Table 3. The commercial antibodies used in this study were listed in the Table 4. The datasets generated and/or analyzed during the current study are not publicly available due to privacy or ethical restrictions, but are available from the corresponding author on reasonable request.

## Abbreviations

ALS: Autophagy lysosome system
CAPN1: Calpain 1
CAPN2: Calpain 2
CAST-L: Calpastatin long isoform
CAST-S: Calpastatin short isoform
CCS: Calpain/calpastatin system
CS: Corticosteroid
ERK1/2: Extracellular signal-regulated kinase 1/2
FAU: 40S ribosomal protein S30
GR1: Glucocorticoid receptor 1
GR2: Glucocorticoid receptor 2
HD: High stocking density
HSP70: Heat-shock protein 70
H2B: Histone 2B
IGF-1: Insulin-like growth factor 1
KLF15: Krüppel-like factor 15
LC3: Microtubule-associated protein 1A/1B-light chain 3
LD: Low stocking density
MR: Mineralocorticoid receptor
MURF1: Muscle RING Finger 1
P62/SQSTM1: Sequestosome-1
qPCR: Quantitative real-time polymerase chain reaction
REDD1: Regulated in development and DNA damage responses 1
TOR: Target of rapamycin
UPS: Ubiquitin proteasome system
4E-BP1: Eukaryotic translation initiation factor 4E-binding protein 1
