# Supplementary material for: Effects of crowding on the three main proteolytic mechanisms of skeletal muscle in rainbow trout (Oncorhynchus mykiss)
Source: BMC Vet Res. 2020 Aug 17;16:294. doi: 10.1186/s12917-020-02518-w (PMC7429773; doi:10.1186/s12917-020-02518-w)

Supplementary Fig. S1

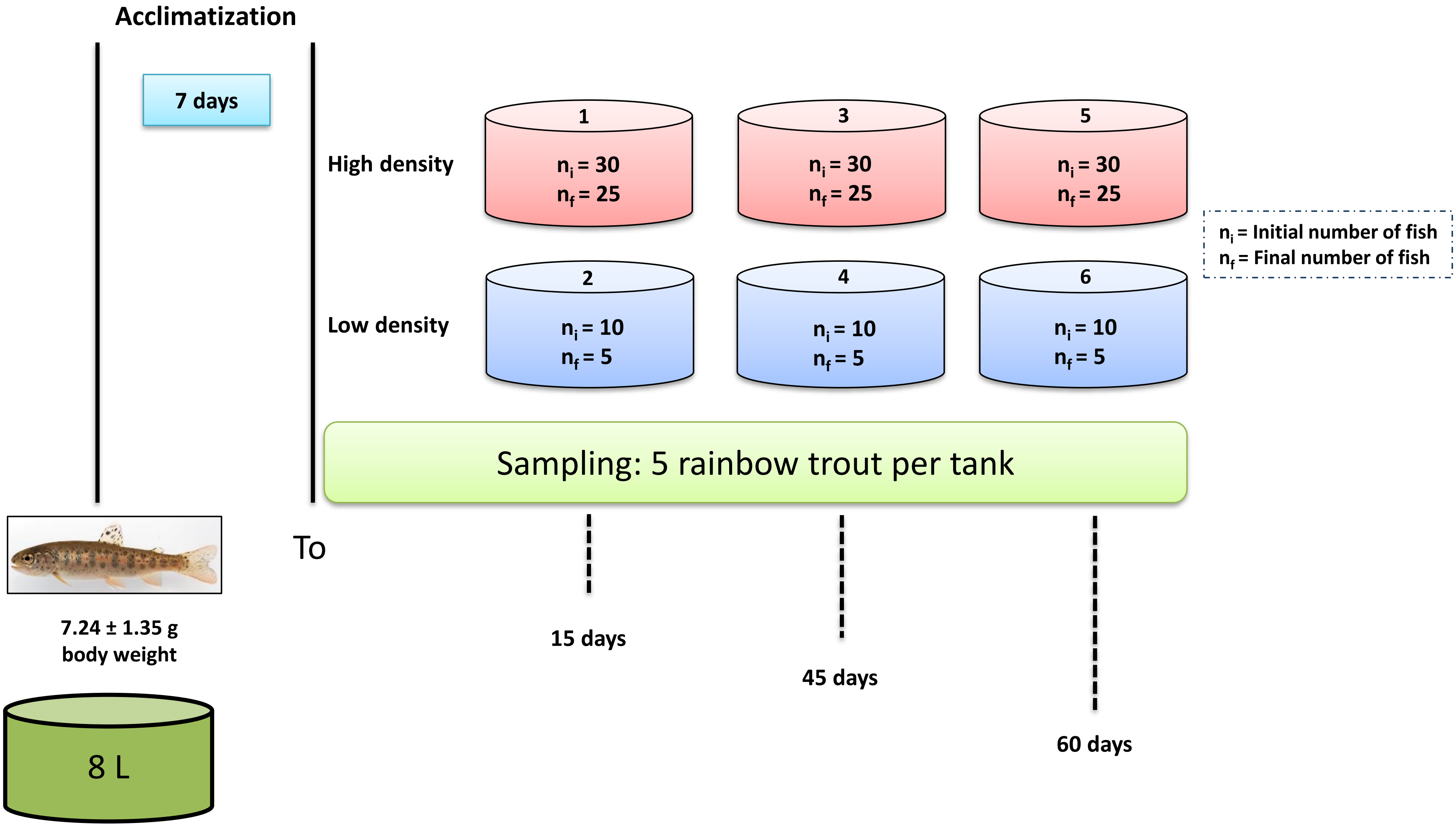

Supplementary Fig. S2

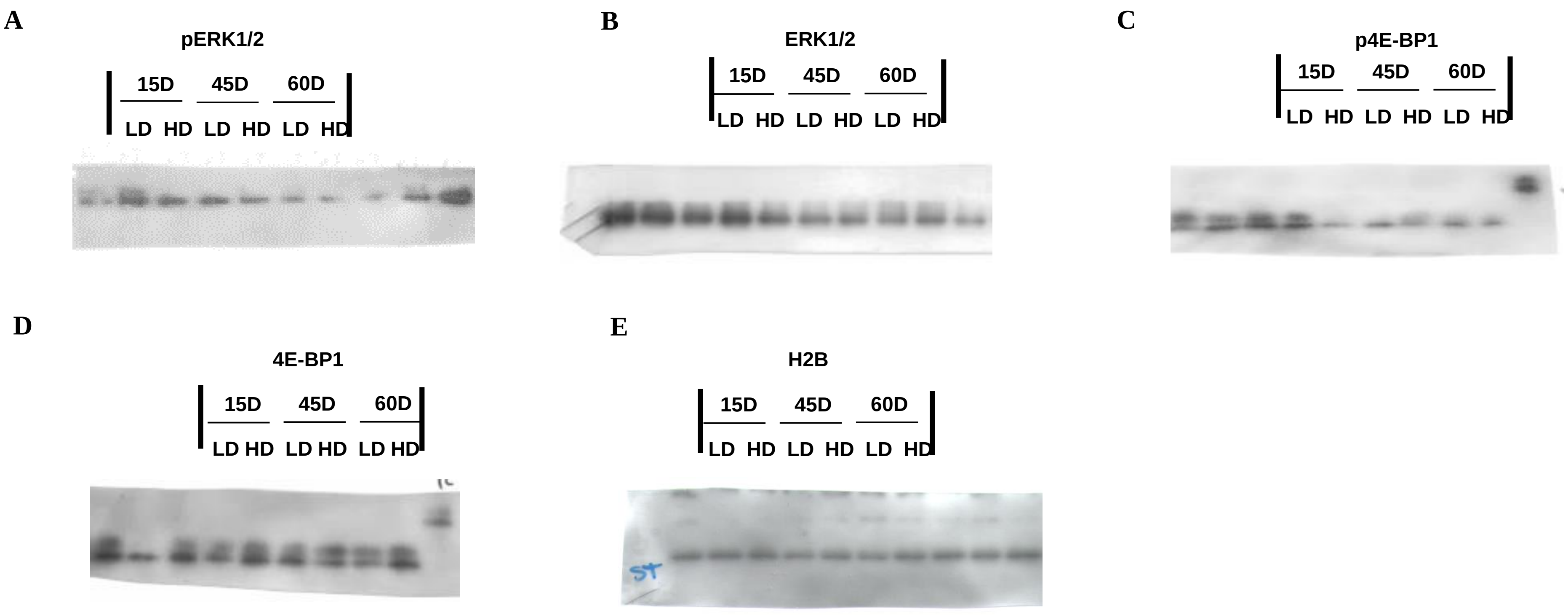

Supplementary Fig. S3

A

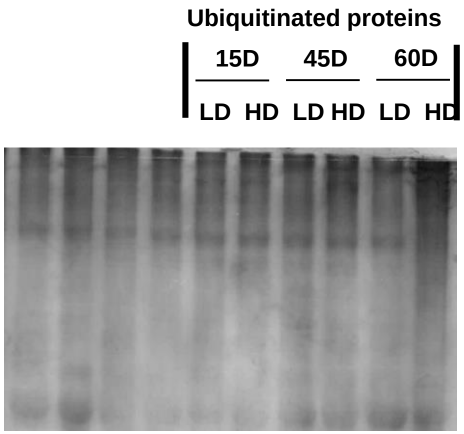

B

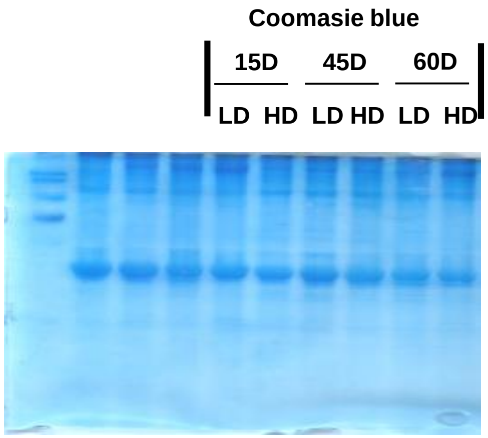

Supplementary Fig. S4

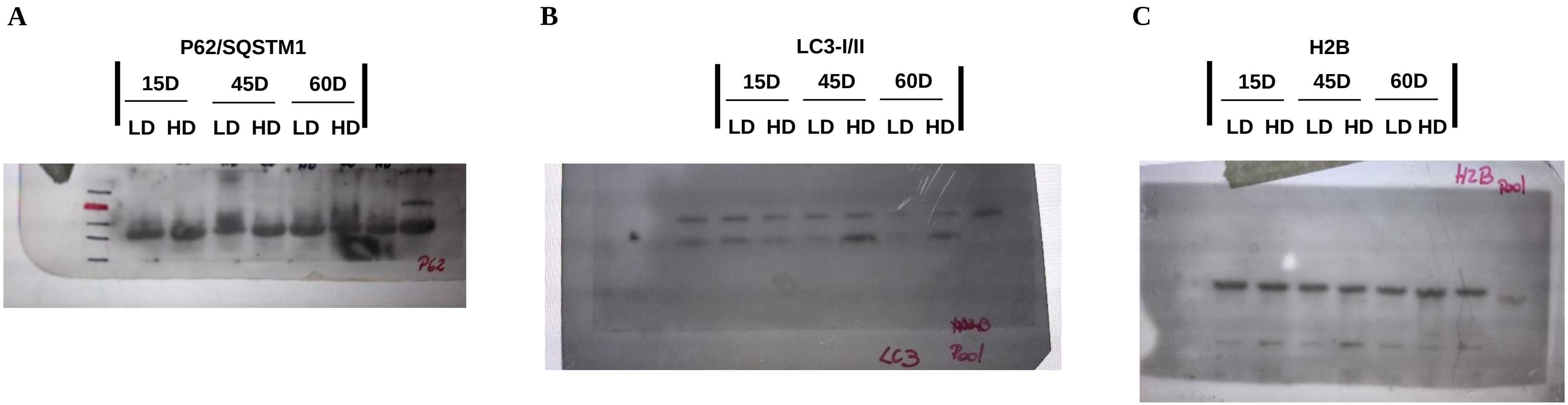

Supplement: Supplementary file 1 — Additional file 1: Figure S1. Graphical abstract of the experimental design. Figure S2. Original western blot images used for growth signaling densitometric analysis. Figure S3. Original western blot and gel images used for ubiquitination densitometric analysis. Figure S4. Original western blot films scanned for autophagy densitometric analysis. [file 12917_2020_2518_MOESM1_ESM.pdf]
